# Patterns of Surgical Prophylaxis Prescribing for Dentoalveolar Procedures in Australian Hospitals: 2016–2022

**DOI:** 10.1111/adj.70047

**Published:** 2026-03-07

**Authors:** Jane Chen, Marietta Taylor, Rodney James, Karin Thursky, Michael McCullough, Leanne Teoh, Courtney Ierano

**Affiliations:** ^1^ Melbourne Dental School The University of Melbourne Melbourne Victoria Australia; ^2^ Royal Melbourne Hospital Guidance Group, Melbourne Health Melbourne Australia; ^3^ National Centre for Antimicrobial Stewardship, Department of Infectious Disease University of Melbourne Melbourne Australia

**Keywords:** antibiotic, antimicrobial, antimicrobial stewardship, dental prescribing, surgical prophylaxis

## Abstract

**Background:**

Antimicrobials are an adjunctive therapy in clinical dentistry. In dentoalveolar surgery, antimicrobials are not routinely required for surgical prophylaxis. This retrospective analysis of the Australian Surgical National Antimicrobial Prescribing Survey (Surgical NAPS) dataset aimed to evaluate the guideline compliance and appropriateness of antimicrobial prescribing for dentoalveolar procedures in Australian hospitals.

**Methodology:**

Deidentified Surgical NAPS data for dentoalveolar procedures (tooth extractions and implant placements) between 2016 and 2022 were extracted. Procedures outside the scope of a general dentist were excluded. Prescribed antimicrobials for surgical prophylaxis, including procedural prophylaxis doses and post‐procedural prescriptions, were assessed for guideline compliance and appropriateness according to the Surgical NAPS algorithm.

**Results:**

1345 surgical episodes with dental procedures were included. This comprised 1077 procedural prophylaxis doses and 555 post‐procedural prescriptions. Of the post‐procedural prescriptions, 478 (86%) were for prophylaxis. Guideline compliance was demonstrated in 35.3% of procedural doses and 14.6% of post‐procedural prescriptions. Rates of appropriateness were 33.5% for procedural doses and 12.6% for post‐procedural prescriptions. Most procedural doses and post‐procedural prescriptions were deemed inappropriate as they were not required (72.5% and 93.0%, respectively).

**Conclusions:**

Suboptimal guideline compliance and appropriateness of antibiotic prescribing for dentoalveolar surgery reinforces the need for antimicrobial stewardship interventions.

## Clinical Relevance

Surgical antibiotic prophylaxis (SAP) is rarely indicated for dentoalveolar procedures. The low compliance with guidelines and suboptimal appropriateness rates of SAP use in routine dentoalveolar surgeries in this study emphasises the critical need for antimicrobial stewardship education and interventions among practitioners in Australian hospitals.

## 1 Introduction

Antimicrobials play an important adjunctive role in the management of a variety of dental conditions. They are commonly prescribed by dental practitioners in both a prophylactic and therapeutic capacity, constituting approximately 10% of total antibiotic prescriptions worldwide [1]. Antimicrobials are indicated as an adjunct to dental treatment in the management of spreading odontogenic infections [2, 3], necrotising periodontal conditions [4, 5] and oral fungal infections [6]. They are recommended prophylactically for the prevention of infective endocarditis [7, 8] and in specific circumstances for dentoalveolar surgical site infections [9, 10].

The global public health threat of antimicrobial resistance (AMR) contributes to morbidity, mortality, financial burden [11] and is estimated to have been associated with 4.7 million deaths in 2021 [12]. Inappropriate antimicrobial prescribing also exposes patients to the unnecessary risk of adverse drug effects such as *Clostridioides difficile* infection and allergic reactions including anaphylaxis, all of which can result in prolonged hospital stays, thereby increasing burden on the healthcare system [13, 14]. It is paramount that dental practitioners uphold principles of antimicrobial stewardship (AMS) adhering to prescribing guidelines and employ clinical judgement when prescribing antimicrobials to balance risks against benefits.

While there are an abundance of studies pertaining to dental prescribing practices in the outpatient and community setting [15, 16, 17, 18, 19, 20], there is a relative scarcity of literature that describes the quality of antimicrobial use for dentoalveolar procedures in the hospital environment. Both third molar extractions [21, 22] and dental implants [23] are commonly performed dentoalveolar procedures in outpatient and hospital settings. Given the surgical nature of these procedures, there is a risk of surgical site infection, and thus, this raises the question of whether surgical antibiotic prophylaxis (SAP) is necessary. Conflicting evidence exists regarding SAP for third molar extractions and dental implant placement [9, 10, 24, 25].

Whilst SAP is not routinely recommended due to relatively low rates of surgical site infections (SSIs) [25, 26], limited evidence supports the prescription of a single preoperative surgical prophylaxis dose to reduce surgical site infections, early implant failures and the prevention of alveolar osteitis. However, this must be considered in the context of a high number needed to treat to prevent these complications [25, 27, 28, 29, 30]. Furthermore, the benefits of SAP may be more apparent in specific patient cohorts (such as immunocompromised individuals or diabetic patients [31]) and for more complex procedures, such as extractions requiring osteotomy [1]. However, research involving these specific patient cohorts is limited and the evidence is of low certainty. Therefore, through retrospective data analysis of the Surgical National Antimicrobial Prescribing Survey (Surgical NAPS) database, the aim of this study was to evaluate the guideline compliance and appropriateness of antimicrobial prescribing for dentoalveolar procedures in Australian hospitals.

## 2 Methods

This study was a retrospective analysis of the Surgical NAPS dataset, assessing the guideline compliance and appropriateness of antibiotic prescribing for dentoalveolar procedures, specifically dental extractions and implants.

### 2.1 Study Population and Characteristics

The Surgical NAPS is a standardised national, multi‐centre online audit that is designed to assist Australian health care facilities to monitor and assess the quality of SAP prescribing during surgical episodes. Participation in the Surgical NAPS is voluntary. The survey collects deidentified data regarding antimicrobials prescribed during surgical episodes and includes both procedural prophylaxis doses and post‐procedural prescriptions. Existing antimicrobial prescriptions were excluded from the analysis.

A surgical episode refers to the set of procedures performed during a single surgical session and included post operative care provided (Table S2. Definitions NAPS). Data collected included patient demographics (e.g., age, sex, weight, geographic location [32], allergies and adverse drug interactions) and other clinical information considered relevant to antimicrobial prescribing (e.g., microbiology results, history of multi‐drug resistant organisms). Details of the antimicrobial prescribed (e.g., drug, dose, frequency, route of administration) and the surgical procedures performed were also recorded. Antimicrobials administered either immediately prior to, or during the surgical procedure, were defined as ‘procedural doses’ and those administered post‐operatively were defined as ‘post‐procedural prescriptions’.

Prescriptions were assessed according to compliance and appropriateness criteria. An antimicrobial was compliant with the Australian national prescribing guidelines (the Australian Therapeutic Guidelines [33]), or with locally endorsed guidelines and if the choice of antimicrobial, its route, dose and frequency, timing and/or duration was deemed compliant for the purposes of this study (Table S3. Compliance with Guidelines). Appropriateness is a more nuanced assessment (Table S4. Appropriateness Definitions) that accounts for the complexity of the patient's medical condition, treatment history and surgical interventions, which may preclude assessment against a standard guideline. Data S1 details the current indications for surgical prophylaxis for dentoalveolar surgical procedures from Therapeutic Guidelines Oral and Dental [33].

### 2.2 Data Collection

Trained pharmacists, nurses, and infectious disease physicians conducted audits of antimicrobial prescribing during surgical episodes. After completing mandatory eLearning training, data were entered into the secure online NAPS platform. This process aimed to ensure standardisation of data collection and compliance and appropriateness assessments. The NAPS support team were available to assist auditors with assessments throughout the auditing process. Facilities could use a convenience sampling method to audit either a targeted surgical specialty or all procedures conducted during a specific time.

### 2.3 Data Inclusion and Exclusion

Antimicrobial prescriptions recorded within ‘dentoalveolar’ surgical episodes were extracted and analysed. More than one surgical procedure can be performed within a surgical episode. Surgical episodes were limited to procedures within the scope of a general dentist, namely dental extractions and implants. Dentoalveolar surgical episodes that included maxillofacial procedures (*n* = 39) such as genioplasty, neck dissection, laryngectomy, malar augmentation, excision of lipoma, osteotomies, free‐flap, tracheostomy and primary repair of cleft palate were excluded as they were considered within the scope of a maxillofacial surgeon. Bone grafts were excluded due to the small sample size (*n* = 1).

### 2.4 Data Analysis

Chi‐squared test was used to compare categorical variables, the two‐sample proportion test was used to compare proportions, and the Wilcoxon rank‐sum test was utilised to compare medians. Statistical analyses were conducted using Stata SE/16.0. Reported *p*‐values were 2‐sided and a *p*‐value of 0.05 was considered statistically significant.

## 3 Results

### 3.1 Demographics and Procedural Characteristics

Between 2016 and 2022, a total of 1345 dentoalveolar surgical episodes were audited. Most episodes comprised extraction‐only (*n* = 1299, 96.6%), with a smaller proportion of implant‐only (*n* = 35, 2.6%) or combined extraction and implant episodes (*n* = 11, 0.8%). The median age of patients was 24 years (IQR: 17–43) and 57% of patients were female. Surgical episodes were predominantly elective (*n* = 1334, 99.2%) and performed within private health facilities (*n* = 1009, 75%). Over half of the surgical episodes were performed in major cities (*n* = 761, 56.6%) (Table 1).

**TABLE 1:** Antimicrobial and patient information by extraction or implant procedure, Surgical NAPS 2016–2022.

	All	Extraction	Implant	Extraction and implant
**Surgical episodes**	**1345**	1299	**35**	**11**
Age in years [median (IQR)]	24 (17–43)	23 (17–40)	58 (45–67)	58 (42–67)
Sex [*n* (%)]				
Female	767 (57.0)	738 (56.8)	23 (65.7)	6 (54.6)
Male	578 (43.0)	561 (43.2)	12 (34.3)	5 (45.5)
Hospital funding				
Private	1009 (75.0)	970 (74.7)	30 (85.7)	9 (81.8)
Public	336 (25.0)	329 (25.3)	5 (14.3)	2 (18.2)
Geographic Location^a^				
Major Cities	761 (56.6)	718 (55.3)	32 (91.4)	11 (100.0)
Inner Regional	119 (8.9)	118 (9.1)	1 (2.9)	0 (0.0)
Outer Regional	460 (34.2)	458 (35.3)	2 (5.7)	0 (0.0)
Remote/Very remote	5 (0.4)	5 (0.4)	0 (0.0)	0 (0.0)
Urgency				
Elective	1334 (99.2)	1288 (99.1)	35 (100.0)	11 (100.0)
Emergency	11 (0.8)	11 (0.9)	0 (0.0)	0 (0.0)
**Procedural prophylaxis doses,** ***n***	**1077**	**1040**	**29**	**8**
Compliance with guidelines				
Compliant with national guidelines	286 (26.6)	274 (26.3)	9 (31.0)	3 (37.5)
Compliant with local guidelines	89 (8.3)	89 (8.6)	0 (0.0)	0 (0.0)
Directed therapy	5 (0.5)	4 (0.4)	0 (0.0)	1 (12.5)
No guidelines available	58 (5.4)	54 (5.2)	4 (13.8)	0 (0.0)
Non‐compliant with guidelines	618 (57.4)	599 (57.6)	15 (51.7)	4 (50.0)
Not assessable	21 (1.9)	20 (1.9)	1 (3.4)	0 (0.0)
Appropriateness				
Appropriate	361 (33.5)	348 (33.4)	10 (34.5)	3 (37.5)
Inappropriate	585 (54.3)	566 (54.4)	14 (48.3)	5 (62.5)
Not assessable/Nil	131 (12.2)	126 (12.1)	5 (17.2)	0 (0.0)
**Post‐procedural prescriptions,** ***n***	**478**	**457**	**17**	**4**
Guideline compliance				
Compliant with national guidelines	22 (4.6)	22 (4.8)	0 (0.0)	0 (0.0)
Compliant with local guidelines	37 (7.7)	37 (8.1)	0 (0.0)	0 (0.0)
Directed therapy	11 (2.3)	6 (1.3)	3 (17.6)	2 (50.0)
No guidelines available	35 (7.3)	35 (7.7)	0 (0.0)	0 (0.0)
Non‐compliant with guidelines	361 (75.5)	345 (75.5)	14 (82.4)	2 (50.0)
Not assessable	12 (2.5)	12 (2.6)	0 (0.0)	0 (0.0)
Appropriateness				
Appropriate	60 (12.6)	60 (13.1)	0 (0.0)	0 (0.0)
Inappropriate	342 (71.5)	321 (70.2)	17 (100.0)	4 (100.0)
Not assessable	76 (15.9)	76 (16.6)	0 (0.0)	0 (0.0)

^a^ See appropriateness definitions at ‘Table S4 …’; Total = sum of procedural doses + post procedural prescriptions; Based on Modified Monash Model categories.

To ensure meaningful subgroup comparisons between extraction‐only and implant‐only procedures, episodes involving both procedures were excluded from the sub‐analysis, as the small sample size (*n* = 11) was insufficient to justify a separate subgroup for analysis.

The extraction‐only cohort had a median age of 23 years (IQR 17–40), and the implant‐only cohort had an older median age of 58 years (IQR 45–67). This difference in patient age was statistically significant (*p* < 0.001). Implant‐only episodes occurred more in major cities compared to extraction‐only episodes (91.4% vs. 55.3%, *p* < 0.001). Of note, 35.3% of extraction‐only episodes, compared to 5.7% of implant‐only, occurred in outer regional regions (*p* < 0.001) (Table 1).

### 3.2 Procedural Prophylaxis Doses

**FIGURE 1:**
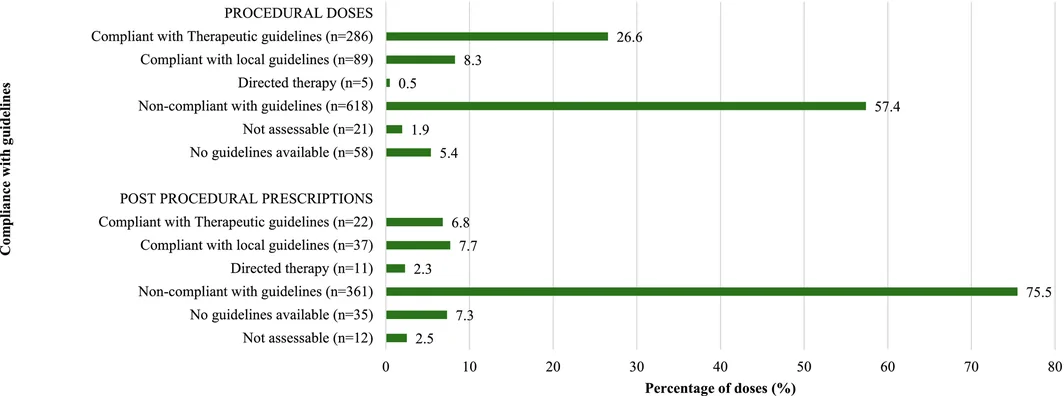
Guideline compliance† of antimicrobials prescribed for dental procedures, Surgical NAPS 2016–2022. † Antibiotic Expert Group. Therapeutic Guidelines: Antibiotic. Version 16. Melbourne: Therapeutic Guidelines Limited; 2019. https://www.tg.org.au/. Procedural doses *n* = 1077, post procedural prescriptions *n* = 478.

Of the 1345 surgical episodes evaluated, 78.7% (*n* = 1059) were administered a procedural prophylaxis antimicrobial dose. No repeat doses were administered. Guideline compliance was low with only 34.8% (*n* = 375) of doses compliant with either national or local guidelines (Table 1, Figure 1).

**FIGURE 2:**
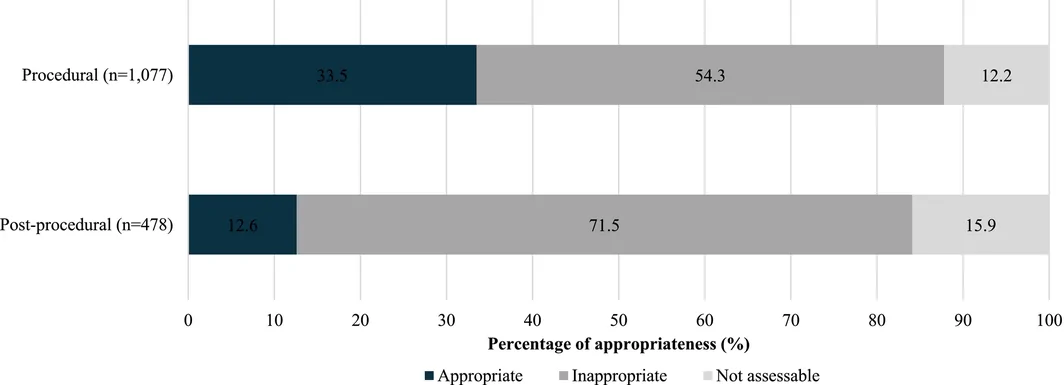
Percentage of antimicrobial appropriateness* of procedural doses and post‐procedural prescriptions for dental procedures, Surgical NAPS 2016–2022. * Refer to Table S4 for surgical antimicrobial appropriateness definitions.

**FIGURE 3:**
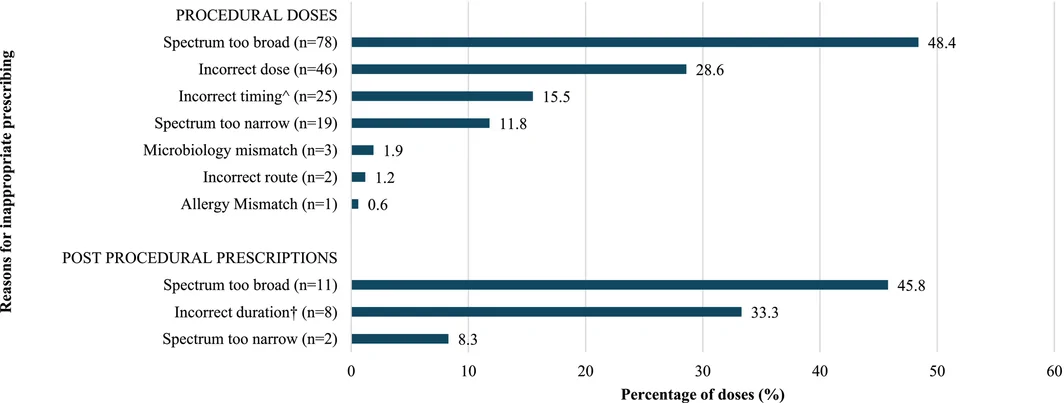
Reasons^**§**^ for inappropriateness* of required antimicrobials for dental procedures, Surgical NAPS 2016–2022. * Refer to Table S4 for appropriateness definitions. An antimicrobial can have more than one reason for inappropriateness. For procedural doses, the total number of doses was *n* = 161, for post‐procedural prescriptions the total number of prescriptions was *n* = 24. ^ Incorrect timing only applies to procedural doses. † Incorrect frequency or incorrect duration only applies to post procedural prescriptions.

The appropriateness of procedural prophylaxis doses was also low, with 33.5% (*n* = 361) assessed as appropriate (Table 1, Figure 2). Among procedural prophylaxis doses deemed inappropriate (54.3%, *n* = 585), the majority (72.5%, *n* = 424) were assessed as ‘not required’, indicating that these doses were administered in clinical scenarios where guidelines do not recommend their use. The remaining inappropriate procedural prophylaxis doses (27.5%, *n* = 161/585) were deemed inappropriate due to reasons such as an excessively broad spectrum (*n* = 78, 48.4%), incorrect dosing (*n* = 46, 28.6%), and incorrect timing (*n* = 25, 15.5%) (Figure 3).

Table 2 provides data on the antimicrobials commonly prescribed for these dental procedures and their rates of appropriateness. Cefazolin was the most common procedural antimicrobial prescribed (74.8%, *n* = 806), with 38.8% deemed appropriate (*n* = 313). Amoxicillin was the second most commonly prescribed antimicrobial (12.3%, *n* = 133), with 18.8% (*n* = 25) deemed appropriate.

**TABLE 2:** Appropriateness^a^ of procedural and post‐procedural prophylaxis antimicrobials prescribed during dental procedures, Surgical NAPS, 2016–2022.

Antimicrobial	Procedural								Post‐procedural							
	Total		Appropriate		Inappropriate		Not assessable		Total		Appropriate		Inappropriate		Not assessable	
	*n*	%	*n*	%	*n*	%	*n*	%	*n*	%	*n*	%	*n*	%	*n*	%
Amoxicillin	133	12.3	26	19.5	107	80.5	—	—	118	24.7	7	5.9	106	89.8	5	4.2
Amoxicillin–clavulanic acid	0	0.0	—	—	—	—	—	—	86	18.0	1	1.2	73	84.9	12	14.0
Ampicillin	54	5.0	3	5.6	51	94.4	—	—	0	0.0	—	—	—	—	—	—
Benzylpenicillin	20	1.9	4	20.0	8	40.0	8	40.0	0	0.0	—	—	—	—	—	—
Cefalexin	13	1.2	1	10.0	9	69.2	—	—	246	51.5	52	21.1	136	55.3	58	23.6
Cefazolin	806	74.8	313	38.8	374	46.4	119	14.8	3	0.6	—	—	—	—	—	—
Clindamycin	16	1.5	6	37.5	10	62.5	—	—	11	2.3	—	—	10	90.9	1	9.1
Metronidazole	29	2.7	5	17.2	22	75.9	2	6.9	12	2.5	—	—	12	100.0	—	—
Other^b^	6	0.6	3	—	3	—	—	—	2	0.4	—	—	2	100.0	—	—
**Total**	**1077**	**100**	**361**	**33.5**	**585**	**54.3**	**131**	**12.2**	**478**	**100**	**60**	**12.6**	**342**	**71.5**	**76**	**15.9**

*Note:* Bold typing is more for clarity (these are the total counts).

^a^ See surgical antimicrobial appropriateness definitions at ‘Table S4’.

^b^ Others = 4 antimicrobials. Procedural: cefalothin, ceftriaxone and lincomycin; post‐procedural: cefaclor.

### 3.3 Post‐Procedural Prescriptions

**FIGURE 4:**
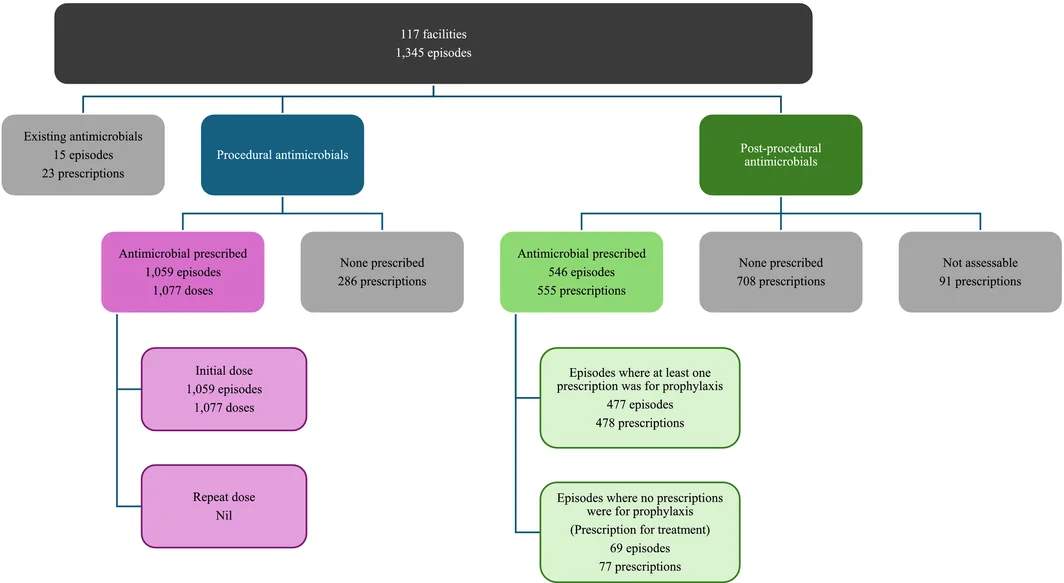
Dental episodes by procedural and post‐procedural antimicrobial characteristics, Surgical NAPS 2016–2022.

Post‐procedural antimicrobials were prescribed in 40.6% (*n* = 546) of surgical episodes. Of these, 87.4% (*n* = 477) of episodes included at least one prescription for prophylaxis (Figure 4). Post‐procedural prophylaxis may be prescribed in the context of profound patient immunocompromise. The proportion of post‐procedural prescriptions assessed as compliant with national and local guidelines was low (12.3%, *n* = 59) (Figure 1). Most post‐procedural prescriptions were deemed non‐compliant (75.5%, *n* = 361) (Table 1).

Approximately one in ten (12.6%, *n* = 60) post‐procedural prescriptions were assessed as appropriate (Table 1) with a further 15.9% (*n* = 76) classified as not assessable. Among post‐procedural prescriptions deemed inappropriate (71.5%, *n* = 342), the most common reason for inappropriateness was when the prescription was deemed ’not required’ (93.0%, *n* = 318).

For the remaining required but inappropriate post‐procedural prescriptions (*n* = 24), the use of an ‘excessively broad spectrum’ antimicrobial was the most common reason for inappropriateness (*n* = 11) (Figure 3). There were fewer reasons for inappropriateness (*n* = 21) than the total number of required inappropriate prescriptions (*n* = 24). This was because the documentation of reasons for inappropriateness was only made mandatory from 2019.

Cefalexin was the most frequently prescribed post‐procedural antimicrobial (51.5%, *n* = 246) with 21.1% of these prescriptions (*n* = 52) deemed appropriate. Amoxicillin represented 24.7% of prescriptions (*n* = 118) and had a very low rate of appropriateness (5.9%, *n* = 7). A small number of other antimicrobials were prescribed post procedurally, with appropriateness ranging from 1.2% to 21.1% (Table 2).

### 3.4 Extraction‐Only Episodes and Implant‐Only Episodes

The extraction‐only and implant‐only episodes accounted for 1299 and 35 surgical episodes respectively. Both subgroups demonstrated low compliance and appropriateness for procedural prophylaxis doses and post‐procedural prescriptions. For extraction only episodes, there was a total of 1040 procedural prophylaxis doses, with a low rate of guideline compliance (34.9%, *n* = 363). Appropriateness of procedural prophylaxis doses was also low, with 33.4% (*n* = 348) assessed as appropriate. Extraction‐only post‐procedural prescriptions comprised 457 prescriptions, with only 12.9% (*n* = 59) deemed as compliant and 13.1% (*n* = 60) deemed as appropriate.

For implant‐only episodes, the compliance and appropriateness from a total of 29 procedural prophylaxis doses was 31.0% (*n* = 9) and 34.5% (*n* = 10) respectively. Post‐procedural prescriptions for implant‐only episodes totalled 17 prescriptions and had no compliant doses (0.0%, *n* = 0) and no doses assessed as appropriate (0.0%, *n* = 0). Within the limitations of these small sample sizes, no statistical significance was found between these subgroups when comparing the compliance and appropriateness in the prescribing of procedural prophylaxis doses or post‐procedural prescriptions (Table 1).

## 4 Discussion

This study is the first comprehensive analysis of procedural and post‐procedural antimicrobial prescribing practices for dentoalveolar surgical episodes (dental extractions and implant placement) in Australian hospitals. This study has identified low rates of guideline compliance and appropriate prophylactic antibiotic use in the context of commonly performed dentoalveolar surgeries. These findings highlight a critical gap in AMS for dentoalveolar surgery, emphasising the need for targeted, evidence‐based strategies to reduce unnecessary prophylaxis and improve prescribing practices in hospital settings.

The predominant reason for inappropriate SAP in the present study was prescribing in situations where there was no clear clinical indication and where evidence or guideline recommendations did not support their use. The routine prescription of SAP for dentoalveolar procedures remains somewhat controversial, with the literature presenting evidence both for and against its use in extractions and implants. This highlights the lack of clarity in clinical practice reinforcing the need for clearer, evidence‐based guidance to inform decision‐making and minimise unnecessary antimicrobial exposure.

Regarding extractions, the literature almost exclusively assesses the effect of SAP for third molar extractions in immunocompetent patients [34], demonstrating varied SSI rates, ranging from 0.1% to 56% [25, 26]. A Cochrane review in 2021 conducted by Lodi et al. [25] that included 23 randomised controlled trials found there was a reduction in SSIs by approximately 66% compared to placebo when SAP was prescribed. The number needed to treat (NNT) was approximately 19. This finding is consistent with other systematic reviews and meta‐analyses [27, 28]. Despite the reduction in SSIs, all studies acknowledged the low certainty of their evidence. There is insufficient high‐quality evidence to support a recommendation with regards to the specific regimen, timing or dosing of SAP [25, 35]. Furthermore, there is a paucity of literature addressing SAP for dental extractions in immunocompromised or systemically compromised groups of patients, such as those with poorly controlled diabetes [31] or patients with transplants [36].

Most studies on SAP for dental implants have focused on implant failure as the primary outcome, with evidence suggesting a protective effect. For example, Roca‐Millan et al. [29] demonstrated that SAP was associated with a 70% reduction of implant failure and Kim et al. [30] reported a 53% relative reduction in implant failure using SAP (NNT = 35).

While infection is a potential cause, it is not the sole factor contributing to implant failure, which complicates interpretation. Studies assessing post‐operative infection (SSI) report mixed findings; a systematic review by Torof et al. [10] demonstrated SAP reduced infection rates (NNT = 32) and reported that implants covered by SAP exhibited a 67% higher survival probability compared to placebo; whereas multiple studies reported no significant difference [37, 38, 39, 40]. High NNT values (25–50) [40, 41, 42, 43] and adverse effects in up to 6% of patients [27, 28] further highlight the limited value of routine SAP in healthy individuals undergoing dentoalveolar procedures. The rate of reported adverse effects is also likely underestimated due to underreporting.

Accordingly, SAP is largely unnecessary for immunocompetent patients undergoing uncomplicated extractions and implant procedures. Thus, the decision to prescribe SAP should be based on individual risk factors and underlying systemic conditions that increase infection risk, rather than being a routine practice. This is a position shared by the Australian national dental guidelines [33] and the Faculty of Dental Practice in the UK [44]. Nevertheless, there is an absence of internationally standardised guidelines for SAP in dental extractions, as most dental prescribing guidelines primarily address antibiotic prophylaxis for infective endocarditis prevention [45, 46, 47, 48]. Furthermore, conflicting recommendations regarding SAP for dental implants are recognised as considerable issues by various professional organisations, including the European Association for Osseointegration and the Spanish Society of Implants [49, 50].

Noncompliance with SAP guidelines is well‐documented across various surgical disciplines [51, 52, 53, 54, 55]. Whilst there is limited literature regarding compliance and appropriateness in dentoalveolar procedures in a hospital setting [56], the findings of the present study align with broader trends across other surgical disciplines. International and Australian data indicate substantial variability in compliance [57], with reported adherence rates ranging from 19.5% to 88% [54, 58, 59, 60, 61, 62], the majority of rates being suboptimal. A recent analysis of antimicrobial prescribing for oral and dental conditions in Australian hospitals from 2013 to 2022 by Teoh et al. [63] reported that guideline compliance differed significantly (*p* < 0.001) between antifungal (80.0%) and antibiotic prescriptions (44.7%). The Surgical NAPS program assesses overall surgical episode appropriateness and reports this annually per surgical procedure group for comparison. Dentoalveolar procedures have consistently reported low rates of appropriateness (35.7%). With the exception of gastrointestinal endoscopic procedures, all other procedure groups reported suboptimal rates of appropriateness (40%–70%), suggesting that this issue is not isolated to dentoalveolar surgery [64]. This highlights the ongoing challenges of inappropriate and/or noncompliant antimicrobial prescribing in perioperative settings. Furthermore, there are varying definitions of compliance and appropriateness within the literature that may render interpretation and comparison difficult between studies.

Consistent with our study, reasons for inappropriate prescribing such as use of SAP when not required or incorrect duration of prophylaxis align with literature across other surgical disciplines. A recent point‐prevalence audit by Jamaluddin et al. [65] described guideline compliance with 32.8% of SAP and appropriateness of 55.2%. The predominant contributor for inappropriateness was incorrect duration (15%) and unnecessary broad‐spectrum coverage (15.6%). Onorato et al. [66] also describe a cohort in which unnecessary prescription of SAP occurred in 10.5% of cases; broad‐spectrum SAP was deemed inappropriate, with the most common reason being the incorrect duration of prophylaxis (76.9%) followed by the prescription of an excessively broad‐spectrum antibiotic (38.5%). Furthermore, a multicentre point prevalence survey conducted in Japan by Morioka et al. [55] found that duration of SAP was only compliant in 43.3% of prescriptions. These findings are not novel and have been consistently documented in the literature over time [59, 67]. Additional reasons for inappropriate prescribing should also be acknowledged, as Enriquez et al. [68] reported that the majority (79.7%) of SAP did not comply with administration timing guidelines. Together, these studies highlight common problems with SAP compliance, particularly around duration, choice, and timing across multiple surgical specialties, including general surgical, orthopaedic, urological, thoracic, plastic, and vascular surgery. While each specialty faces its own unique challenges, the consistency of these findings points to the need for both system‐wide AMS strategies and specialty‐tailored interventions to improve SAP prescribing practices.

Numerous factors contribute to suboptimal adherence to SAP guidelines in clinical practice. These include surgeons' knowledge, familiarity and agreement with SAP guidelines, and their accessibility, logistical barriers affecting the timing of administration, and limited engagement by senior surgeons in AMS protocol development and implementation [69, 70]. Disagreement between surgical and anaesthetic staff regarding responsibility for SAP decision‐making further complicates guideline compliance. Furthermore, there has been a rise in defensive medical practices that may influence prescribing, which is the perception that providing additional antimicrobial doses may serve as a safeguard against medicolegal action in cases where a patient develops an infection [71, 72].

This study has several limitations. As participation in the Surgical NAPS program is voluntary, the results may not be generalisable to all hospitals. It is possible that hospitals with well‐established AMS programs may be more inclined to participate, with a potential for underestimation of noncompliance and inappropriateness rates. Appropriateness assessments inevitably involve an element of subjectivity. The development of standardised assessment criteria, auditor evaluations through online training and the availability of support from the NAPS team aims to minimise such subjectivity. Additionally, audit data were collected by medical professionals, nurses or pharmacists, which may impact the ability to assess the appropriateness of dentoalveolar procedures due to a lack of familiarity with these procedures. The small dataset of implant‐only cases limits the generalisability of these findings. Lastly, clinical outcomes, including SSIs and antibiotic‐related adverse events, were not collected within this dataset. Future research incorporating post‐procedural review would be beneficial in understanding the impact of SAP prescribing practices on patient outcomes.

Nonetheless, the findings of this study provide a foundational analysis of SAP compliance and appropriateness in dentoalveolar procedures and can support the development of further resources to improve prescribing practices, such as targeted audit and feedback, clinician education and guideline development and implementation. Importantly, audit and feedback is central to many AMS strategies and, despite being labour‐intensive, provides the foundation for meaningful education and targeted behavioural interventions [73, 74]. Interventional strategies targeting dental antibiotic prescribing, such as audit and feedback, guideline implementation and education, have shown promise in improving compliance and reducing unnecessary use [75, 76]. These strategies highlight the potential for AMS programs to mitigate antimicrobial overuse, improve patient safety and limit the further development of AMR.

## 5 Conclusion

This retrospective analysis of the Surgical NAPS dataset demonstrated suboptimal rates of guideline compliance and appropriateness for procedural and post‐procedural antimicrobials for dentoalveolar surgery in Australian hospitals. Targeted AMS strategies, particularly audit and feedback, alongside standardised, accessible guidelines for both surgeons and dentists are urgently needed to optimise antimicrobial use, strengthen compliance with evidence‐based practices, and improve patient safety.

## Author Contributions

**Jane Chen:** conceptualisation, methodology, writing – original draft, writing – review and editing. **Marietta Taylor:** conceptualisation, validation, formal analysis, writing – review and editing, visualisation. **Rodney James:** conceptualisation, data curation, writing – review and editing. **Karin Thursky:** conceptualisation, resources, writing – review and editing. **Michael McCullough:** conceptualisation, writing – review and editing. **Leanne Teoh:** conceptualisation, resources, methodology, writing – review and editing, project administration, supervision. **Courtney Ierano:** conceptualisation, methodology, investigation, writing – review and editing, supervision.

## Funding

The authors have nothing to report. L.T. was supported by a National Health and Medical Research Council Investigator Grant (2016647).

## Disclosure

*Generative AI Declaration*: The authors declare that no AI tools were used in the preparation or drafting of this manuscript.

## Ethics Statement

The data were de‐identified at both patient and hospital levels. The Surgical NAPS dataset has been approved for use in research by the Melbourne Health Human Research Ethic Committee (HREC/74195/MH‐2022: The National Antimicrobial Prescribing Survey Data for Research Use).

## Conflicts of Interest

The authors declare no conflicts of interest.

## Supporting information


**Data S1:** Supporting Information.

## Data Availability

The data that support the findings of this study are available from the corresponding author upon reasonable request.

## References

[1] A. Buonavoglia , P. Leone , A. G. Solimando , et al., “Antibiotics or No Antibiotics, That Is the Question: An Update on Efficient and Effective Use of Antibiotics in Dental Practice,” Antibiotics 10, no. 5 (2021): 550. 34065113 10.3390/antibiotics10050550 PMC8151289

[2] J. R. Martins , O. L. Chagas, Jr. , B. D. Velasques , A. N. Bobrowski , M. B. Correa , and M. A. Torriani , “The Use of Antibiotics in Odontogenic Infections: What Is the Best Choice? A Systematic Review,” Journal of Oral and Maxillofacial Surgery 75, no. 12 (2017): 2606. 10.1016/j.joms.2017.08.017 28893540

[3] L. Teoh , M. C. Cheung , S. Dashper , R. James , and M. J. McCullough , “Oral Antibiotic for Empirical Management of Acute Dentoalveolar Infections—A Systematic Review,” Antibiotics (Basel) 10, no. 3 (2021): 240. 33670844 10.3390/antibiotics10030240 PMC7997333

[4] D. Herrera , B. Alonso , L. de Arriba , I. Santa Cruz , C. Serrano , and M. Sanz , “Acute Periodontal Lesions,” Periodontology 2000 65, no. 1 (2000): 149–177. 10.1111/prd.12022 24738591

[5] H. Ahmadi , A. Ebrahimi , and F. Ahmadi , “Antibiotic Therapy in Dentistry,” International Journal of Dentistry 2021 (2021): 6667624. 33574843 10.1155/2021/6667624 PMC7861949

[6] D. R. Telles , N. Karki , and M. W. Marshall , “Oral Fungal Infections: Diagnosis and Management,” Dental Clinics of North America 61, no. 2 (2017): 319–349. 28317569 10.1016/j.cden.2016.12.004

[7] A. E. Gross , T. Khouja , S. A. Rowan , and K. J. Suda , “Unnecessary Antibiotic Prescribing in Dental Practices and Associated Adverse Effects,” Current Infectious Disease Reports 23 (2021): 1–6.

[8] F. Sperotto , K. France , M. Gobbo , et al., “Antibiotic Prophylaxis and Infective Endocarditis Incidence Following Invasive Dental Procedures: A Systematic Review and Meta‐Analysis,” JAMA Cardiology 9 (2024): 599. 38581643 10.1001/jamacardio.2024.0873 PMC10999003

[9] T. Khouja , E. Kennedy , and K. J. Suda , “Antibiotic Prophylaxis for Tooth Extractions and Dental Implants, A Narrative Review,” Current Infectious Disease Reports 25, no. 5 (2023): 87–99.

[10] E. Torof , H. Morrissey , and P. A. Ball , “The Role of Antibiotic Use in Third Molar Tooth Extractions: A Systematic Review and Meta‐Analysis,” Medicina (Kaunas) 59, no. 3 (2023): 422. 36984426 10.3390/medicina59030422 PMC10054344

[11] Bank W , Drug‐Resistant Infections: A Threat to Our Economic Future (World Bank, 2017).

[12] G. B. D. A. R. Collaborators , “Global Burden of Bacterial Antimicrobial Resistance 1990–2021: A Systematic Analysis With Forecasts to 2050,” Lancet 404, no. 10459 (2024): 1199–1226. 39299261 10.1016/S0140-6736(24)01867-1 PMC11718157

[13] T. M. Paumier , “Appropriate Antibiotic Use in Dentistry: A Review of the Literature and Clinical Recommendations,” General Dentistry 72, no. 1 (2024): 27–33. 38117638

[14] C. Dammling , E. M. Gilmartin , S. Abramowicz , and B. Kinard , “Indications for Antibiotic Prophylaxis for Dentoalveolar Procedures,” Dental Clinics of North America 68, no. 1 (2024): 99–111. 37951640 10.1016/j.cden.2023.07.004

[15] L. Teoh , K. Stewart , R. J. Marino , and M. J. McCullough , “Part 1. Current Prescribing Trends of Antibiotics by Dentists in Australia From 2013 to 2016,” Australian Dental Journal 63 (2018): 329–337. 10.1111/adj.12622 29754452

[16] L. Sbricoli , G. Grisolia , E. Stellini , C. Bacci , M. Annunziata , and E. Bressan , “Antibiotic‐Prescribing Habits in Dentistry: A Questionnaire‐Based Study,” Antibiotics (Basel) 13, no. 2 (2024): 189. 38391575 10.3390/antibiotics13020189 PMC10886335

[17] W. Thompson , L. Teoh , C. C. Hubbard , et al., “Patterns of Dental Antibiotic Prescribing in 2017: Australia, England, United States, and British Columbia (Canada),” Infection Control and Hospital Epidemiology 43, no. 2 (2022): 191–198. 33818323 10.1017/ice.2021.87 PMC9044466

[18] F. Tousi , M. Al Haroni , S. A. Lie , and B. Lund , “Antibiotic Prescriptions Among Dentists Across Norway and the Impact of COVID‐19 Pandemic,” BMC Oral Health 23, no. 1 (2023): 649. 37684614 10.1186/s12903-023-03380-6 PMC10492408

[19] A. M. Murphy , U. C. Patel , G. M. Wilson , and K. J. Suda , “Prevalence of Unnecessary Antibiotic Prescriptions Among Dental Visits,” Infection Control and Hospital Epidemiology 2024 (2019): 1–10. 10.1017/ice.2024.13 38374683

[20] C. Loffler and F. Bohmer , “The Effect of Interventions Aiming to Optimise the Prescription of Antibiotics in Dental Care‐A Systematic Review,” PLoS One 12, no. 11 (2017): e0188061. 29136646 10.1371/journal.pone.0188061 PMC5685629

[21] D. L. M. Broers , L. Dubois , J. de Lange , N. Su , and A. de Jongh , “Reasons for Tooth Removal in Adults: A Systematic Review,” International Dental Journal 72, no. 1 (2022): 52–57. 33648772 10.1016/j.identj.2021.01.011 PMC9275356

[22] T. B. Dodson and S. M. Susarla , “Impacted Wisdom Teeth,” BMJ Clinical Evidence (2014): 1302. PMC4148832 25170946

[23] H. W. Elani , J. R. Starr , J. D. Da Silva , and G. O. Gallucci , “Trends in Dental Implant Use in the U.S., 1999‐2016, and Projections to 2026,” Journal of Dental Research 97, no. 13 (2018): 1424–1430. 30075090 10.1177/0022034518792567 PMC6854267

[24] M. V. Cuevas‐Gonzalez , J. C. Cuevas‐Gonzalez , L. F. Espinosa‐Cristobal , et al., “Use or Abuse of Antibiotics as Prophylactic Therapy in Oral Surgery: A Systematic Review,” Medicine (Baltimore) 102, no. 37 (2023): e35011. 37713865 10.1097/MD.0000000000035011 PMC10508532

[25] G. Lodi , L. Azzi , E. M. Varoni , et al., “Antibiotics to Prevent Complications Following Tooth Extractions,” Cochrane Database of Systematic Reviews 2, no. 2 (2021): CD003811. 33624847 10.1002/14651858.CD003811.pub3 PMC8094158

[26] E. Kostares , G. Kostare , M. Kostares , A. Tsakris , and M. Kantzanou , “Prevalence of Surgical Site Infections Following Extraction of Impacted Mandibular Third Molars: A Systematic Review and Meta‐Analysis,” Journal of Stomatology, Oral and Maxillofacial Surgery 126, no. 1 (2024): 101995. 39084557 10.1016/j.jormas.2024.101995

[27] E. Ramos , J. Santamaria , G. Santamaria , L. Barbier , and I. Arteagoitia , “Do Systemic Antibiotics Prevent Dry Socket and Infection After Third Molar Extraction? A Systematic Review and Meta‐Analysis,” Oral Surgery, Oral Medicine, Oral Pathology, Oral Radiology 122, no. 4 (2016): 403–425. 27499028 10.1016/j.oooo.2016.04.016

[28] O. Camps‐Font , H. Sabado‐Bundo , J. Toledano‐Serrabona , N. Valmaseda‐de‐la‐Rosa , R. Figueiredo , and E. Valmaseda‐Castellon , “Antibiotic Prophylaxis in the Prevention of Dry Socket and Surgical Site Infection After Lower Third Molar Extraction: A Network Meta‐Analysis,” International Journal of Oral and Maxillofacial Surgery 53, no. 1 (2024): 57–67. 37612199 10.1016/j.ijom.2023.08.001

[29] E. Roca‐Millan , A. Estrugo‐Devesa , A. Merlos , E. Jane‐Salas , T. Vinuesa , and J. Lopez‐Lopez , “Systemic Antibiotic Prophylaxis to Reduce Early Implant Failure: A Systematic Review and Meta‐Analysis,” Antibiotics (Basel) 10, no. 6 (2021): 698. 34200841 10.3390/antibiotics10060698 PMC8230529

[30] A. S. Kim , N. Abdelhay , L. Levin , J. D. Walters , and M. P. Gibson , “Antibiotic Prophylaxis for Implant Placement: A Systematic Review of Effects on Reduction of Implant Failure,” British Dental Journal 228, no. 12 (2020): 943–951. 32591710 10.1038/s41415-020-1649-9 PMC7319948

[31] M. Sykara , P. Maniatakos , A. Tentolouris , I. K. Karoussis , and N. Tentolouris , “The Necessity of Administrating Antibiotic Prophylaxis to Patients With Diabetes Mellitus Prior to Oral Surgical Procedures—A Systematic Review,” Diabetes and Metabolic Syndrome: Clinical Research and Reviews 16, no. 10 (2022): 102621. 10.1016/j.dsx.2022.102621 36183455

[32] ABS , Australian Statistical Geography Standard (ASGS): Volume 5–Remoteness (Australian Bureau of Statistics Canberra, 2018), https://www.abs.gov.au/ausstats/abs@.nsf/mf/1270.0.55.005.

[33] Therapeutic Guidelines Oral and Dental. Version 3 (Therapeutic Guidelines Pty Ltd, 2019).

[34] S. Marchionni , P. Toti , A. Barone , U. Covani , and M. Esposito , “The Effectiveness of Systemic Antibiotic Prophylaxis in Preventing Local Complications After Tooth Extraction. A Systematic Review. Eur,” Journal of Oral Implantology 10, no. 2 (2017): 127–132. 28555203

[35] S. G. M. Falci , E. L. Galvao , G. M. de Souza , I. A. Fernandes , M. R. F. Souza , and E. A. Al‐Moraissi , “Do Antibiotics Prevent Infection After Third Molar Surgery? A Network Meta‐Analysis,” International Journal of Oral and Maxillofacial Surgery 51, no. 9 (2022): 1226–1236. 35527115 10.1016/j.ijom.2022.04.001

[36] J. Guggenheimer , B. Eghtesad , and D. J. Stock , “Dental Management of the (Solid) Organ Transplant Patient,” Oral Surgery, Oral Medicine, Oral Pathology, Oral Radiology, and Endodontics 95, no. 4 (2003): 383–389. 12686921 10.1067/moe.2003.150

[37] I. Khouly , R. S. Braun , and L. Chambrone , “Antibiotic Prophylaxis May Not Be Indicated for Prevention of Dental Implant Infections in Healthy Patients. A Systematic Review and Meta‐Analysis,” Clinical Oral Investigations 23, no. 4 (2019): 1525–1553. 30824982 10.1007/s00784-018-2762-x

[38] F. Rodriguez Sanchez , C. Rodriguez Andres , and I. Arteagoitia , “Which Antibiotic Regimen Prevents Implant Failure or Infection After Dental Implant Surgery? A Systematic Review and Meta‐Analysis,” Journal of Cranio‐Maxillo‐Facial Surgery 46, no. 4 (2018): 722–736. 29550218 10.1016/j.jcms.2018.02.004

[39] J. Park , M. Tennant , L. J. Walsh , and E. Kruger , “Is There a Consensus on Antibiotic Usage for Dental Implant Placement in Healthy Patients?,” Australian Dental Journal 63, no. 1 (2018): 25–33. 28543332 10.1111/adj.12535

[40] J. Ata‐Ali , F. Ata‐Ali , and F. Ata‐Ali , “Do Antibiotics Decrease Implant Failure and Postoperative Infections? A Systematic Review and Meta‐Analysis,” International Journal of Oral and Maxillofacial Surgery 43, no. 1 (2014): 68–74. 23809986 10.1016/j.ijom.2013.05.019

[41] M. Esposito , M. G. Grusovin , and H. V. Worthington , “Interventions for Replacing Missing Teeth: Antibiotics at Dental Implant Placement to Prevent Complications,” Cochrane Database of Systematic Reviews 2013, no. 7 (2013): CD004152. 23904048 10.1002/14651858.CD004152.pub4 PMC6786879

[42] B. Lund , M. Hultin , S. Tranaeus , A. Naimi‐Akbar , and B. Klinge , “Complex Systematic Review ‐ Perioperative Antibiotics in Conjunction With Dental Implant Placement,” Clinical Oral Implants Research 26, no. Suppl 11 (2015): 1–14. 10.1111/clr.12637 26080862

[43] A. Singh Gill , H. Morrissey , and A. Rahman , “A Systematic Review and Meta‐Analysis Evaluating Antibiotic Prophylaxis in Dental Implants and Extraction Procedures,” Medicina (Kaunas, Lithuania) 54, no. 6 (2018): 95. 30513764 10.3390/medicina54060095 PMC6306745

[44] N. Palmer , N. Seoudi , M. Ide , C. Randall , L. Hyland , and A. Patrick , Antimicrobial Prescribing in Dentistry: Good Practice Guidelines (Royal College of Surgeons of England, 2020).

[45] W. R. Wilson , M. Gewitz , P. B. Lockhart , et al., “Prevention of Viridans Group Streptococcal Infective Endocarditis: A Scientific Statement From the American Heart Association,” Circulation 143, no. 20 (2021): e963–e978. 33853363 10.1161/CIR.0000000000000969

[46] National Institute for Health and Care Excellence: Guidelines. Prophylaxis Against Infective Endocarditis: Antimicrobial Prophylaxis Against Infective Endocarditis in Adults and Children Undergoing Interventional Procedures (National Institute for Health and Care Excellence (NICE) Copyright NICE 2018, 2016). 32134603

[47] Excellence NIfHaC , Prophylaxis Against Infective Endocarditis: Antimicrobial Prophylaxis Against Infective Endocarditis in Adults and Children Undergoing Interventional Procedures (NICE, 2008), https://www.nice.org.uk/guidance/cg64. 21656971

[48] V. Delgado , N. Ajmone Marsan , S. de Waha , et al., “2023 ESC Guidelines for the Management of Endocarditis,” European Heart Journal 44, no. 39 (2023): 3948–4042. 37622656 10.1093/eurheartj/ehad193

[49] A. O. Salgado‐Peralvo , A. Garcia‐Sanchez , N. Kewalramani , et al., “Consensus Report on Preventive Antibiotic Therapy in Dental Implant Procedures: Summary of Recommendations From the Spanish Society of Implants,” Antibiotics (Basel) 11, no. 5 (2022): 655. 35625298 10.3390/antibiotics11050655 PMC9138127

[50] A. Caiazzo , L. Canullo , and P. Pesce , “Consensus Report by the Italian Academy of Osseointegration on the Use of Antibiotics and Antiseptic Agents in Implant Surgery,” International Journal of Oral & Maxillofacial Implants 36, no. 1 (2021): 103–105. 33600529 10.11607/jomi.8264

[51] M. Piscaglia , D. Martin Sierra , A. Huelva Millan , M. T. Garcia Poo , J. Rodriguez Bano , and M. D. Del Toro Lopez , “Adequacy and Implications of Antimicrobial Prophylaxis for Elective Surgeries in a Tertiary Hospital: A Cross Sectional and Retrospective Cohort Study (ADEQUAP),” Antimicrobial Resistance and Infection Control 14, no. 1 (2025): 82. 40619410 10.1186/s13756-025-01601-x PMC12232766

[52] S. Goncalves , N. Mohammedi , F. Antonini , et al., “Compliance With Antimicrobial Stewardship Guidelines in Surgery: An Observational, Multidisciplinary, Cohort Study,” World Journal of Emergency Surgery: WJES 20, no. 1 (2025): 63. 40684205 10.1186/s13017-025-00636-0 PMC12275391

[53] S. M. Cabral , A. D. Harris , S. E. Cosgrove , L. S. Magder , P. D. Tamma , and K. E. Goodman , “Adherence to Antimicrobial Prophylaxis Guidelines for Elective Surgeries Across 825 US Hospitals, 2019‐2020,” Clinical Infectious Diseases 76, no. 12 (2023): 2106–2115. 36774539 10.1093/cid/ciad077

[54] S. Celik Ekinci , E. Yenilmez , G. Akengin Ocal , et al., “Surgical Antimicrobial Prophylaxis Compliance in Turkey: Data From the Prospective, Observational, Multicenter Survey Including 7,978 Surgical Patients,” Surgical Infections 25, no. 3 (2024): 231–239. 38588521 10.1089/sur.2023.243

[55] H. Morioka , Y. Koizumi , T. Watariguchi , et al., “Surgical Antimicrobial Prophylaxis in Japanese Hospitals: Real Status and Challenges,” Journal of Infection and Chemotherapy 30, no. 7 (2024): 626–632. 38272262 10.1016/j.jiac.2024.01.013

[56] G. Patri , C. M. Savani , L. S. L. Colaco , S. Sourabh , L. Priya , and P. Meston , “Impact of Antibiotic Stewardship on Infection Rates in Oral Surgery Procedures: A Descriptive Study,” Oral Surgery, Oral Medicine, Oral Pathology, Oral Radiology 140, no. 2 (2025): e29–e34. 40335404 10.1016/j.oooo.2025.02.020

[57] C. Ierano , K. Thursky , C. Marshall , et al., “Appropriateness of Surgical Antimicrobial Prophylaxis Practices in Australia,” JAMA Network Open 2, no. 11 (2019): e1915003. 31702804 10.1001/jamanetworkopen.2019.15003 PMC6902799

[58] Y. M. Alahmadi , R. H. Alharbi , A. K. Aljabri , F. S. Alofi , O. A. Alshaalani , and B. H. Alssdi , “Adherence to the Guidelines for Surgical Antimicrobial Prophylaxis in a Saudi Tertiary Care Hospital,” Journal of Taibah University Medical Sciences 15, no. 2 (2020): 136–141. 32368210 10.1016/j.jtumed.2020.01.005 PMC7184216

[59] S. Mousavi , E. Zamani , and F. Bahrami , “An Audit of Perioperative Antimicrobial Prophylaxis: Compliance With the International Guidelines,” Journal of Research in Pharmacy Practice 6, no. 2 (2017): 126–129. 28616437 10.4103/jrpp.JRPP_16_164 PMC5463548

[60] C. E. Tourmousoglou , E. Yiannakopoulou , V. Kalapothaki , et al., “Adherence to Guidelines for Antibiotic Prophylaxis in General Surgery: A Critical Appraisal,” Journal of Antimicrobial Chemotherapy 61, no. 1 (2008): 214–218. 17999981 10.1093/jac/dkm406

[61] C. Hohmann , C. Eickhoff , R. Radziwill , and M. Schulz , “Adherence to Guidelines for Antibiotic Prophylaxis in Surgery Patients in German Hospitals: A Multicentre Evaluation Involving Pharmacy Interns,” Infection 40, no. 2 (2012): 131–137. 22002734 10.1007/s15010-011-0204-7

[62] P. Dias , A. Patel , W. Rook , et al., “Contemporary Use of Antimicrobial Prophylaxis for Surgical Patients: An Observational Cohort Study,” European Journal of Anaesthesiology 39, no. 6 (2022): 533–539. 34738963 10.1097/EJA.0000000000001619

[63] L. Teoh , M. Taylor , C. Ierano , M. McCullough , K. Thursky , and R. James , “Appropriateness of Antimicrobial Prescribing for Oral and Dental Conditions in Australian Hospitals: 2013 to 2022,” Journal of Dentistry 148 (2024): 105241. 39009335 10.1016/j.jdent.2024.105241

[64] Stewardship RMHaNCfA. Surgical prophylaxis prescribing in Australian hospitals. Results of the , Surgical National Antimicrobial Prescribing Survey (Department of Health and Aged Care, 2022), 2024.

[65] N. A. H. Jamaluddin , P. Periyasamy , C. L. Lau , et al., “Assessment of Antimicrobial Prescribing Patterns, Guidelines Compliance, and Appropriateness of Antimicrobial Prescribing in Surgical‐Practice Units: Point Prevalence Survey in Malaysian Teaching Hospitals,” Frontiers in Pharmacology 15 (2024): 1381843. 38720771 10.3389/fphar.2024.1381843 PMC11076853

[66] L. Onorato , M. Macera , C. Curatolo , et al., “Impact of a Persuasive Antimicrobial Stewardship Programme on the Appropriateness of Surgical Antimicrobial Prophylaxis in a Tertiary Care Hospital in Southern Italy,” Journal of Global Antimicrobial Resistance 39 (2024): 122–127. 39278461 10.1016/j.jgar.2024.09.003

[67] A. Abdel‐Aziz , A. El‐Menyar , H. Al‐Thani , et al., “Adherence of Surgeons to Antimicrobial Prophylaxis Guidelines in a Tertiary General Hospital in a Rapidly Developing Country,” Advances in Pharmacological Sciences 2013 (2013): 842593. 24454349 10.1155/2013/842593 PMC3885161

[68] K. Enriquez , S. Raouf , J. Shakpeh , M. Niescierenko , and F. Mayah‐Toto , “Surgical Antimicrobial Prophylaxis Among Surgical Patients: Results From a Retrospective Observational Study at a Public Hospital in Liberia,” BMJ Open 12, no. 7 (2022): e059018. 10.1136/bmjopen-2021-059018 PMC9280871 35831053

[69] J. Broom , A. Broom , C. Anstey , et al., “Barriers‐Enablers‐Ownership Approach: A Mixed Methods Analysis of a Social Intervention to Improve Surgical Antibiotic Prescribing in Hospitals,” BMJ Open 11, no. 5 (2021): e046685. 10.1136/bmjopen-2020-046685 PMC8112423 33972342

[70] C. Ierano , K. Thursky , T. Peel , A. Rajkhowa , C. Marshall , and D. Ayton , “Influences on Surgical Antimicrobial Prophylaxis Decision Making by Surgical Craft Groups, Anaesthetists, Pharmacists and Nurses in Public and Private Hospitals,” PLoS One 14, no. 11 (2019): e0225011. 31725771 10.1371/journal.pone.0225011 PMC6855473

[71] R. S. Ng and C. P. Chong , “Surgeons' Adherence to Guidelines for Surgical Antimicrobial Prophylaxis ‐ a Review,” Australasian Medical Journal 5, no. 10 (2012): 534–540. 23173017 10.4066/AMJ.2012.1312 PMC3494825

[72] S. Hassan , V. Chan , J. Stevens , and I. Stupans , “Factors That Influence Adherence to Surgical Antimicrobial Prophylaxis (SAP) Guidelines: A Systematic Review,” Systematic Reviews 10, no. 1 (2021): 29. 33453730 10.1186/s13643-021-01577-w PMC7811740

[73] G. W. Chung , J. E. Wu , C. L. Yeo , D. Chan , and L. Y. Hsu , “Antimicrobial Stewardship: A Review of Prospective Audit and Feedback Systems and an Objective Evaluation of Outcomes,” Virulence 4, no. 2 (2013): 151–157. 23302793 10.4161/viru.21626 PMC3654615

[74] J. Satterfield , A. R. Miesner , and K. M. Percival , “The Role of Education in Antimicrobial Stewardship,” Journal of Hospital Infection 105, no. 2 (2020): 130–141. 32243953 10.1016/j.jhin.2020.03.028

[75] L. Teoh , C. Loffler , M. Mun , et al., “A Systematic Review of Dental Antibiotic Stewardship Interventions,” Community Dentistry and Oral Epidemiology 53 (2024): 245–255. 39400429 10.1111/cdoe.13009 PMC12064870

[76] L. Teoh , W. Thompson , and K. Suda , “Antimicrobial Stewardship in Dental Practice,” Journal of the American Dental Association (1939) 151, no. 8 (2020): 589–595. 32718488 10.1016/j.esmoop.2020.04.023

