# Supplementary material for: Patterns of Surgical Prophylaxis Prescribing for Dentoalveolar Procedures in Australian Hospitals: 2016–2022
Source: Aust Dent J. 2026 Mar 7;71(3):231–41. doi: 10.1111/adj.70047 (PMC13506666; doi:10.1111/adj.70047)
Supplement: Supplementary file 1 [file ADJ-71-231-s001.docx]

## Appendix Data 1: Current indications for surgical prophylaxis for dentoalveolar surgical procedures

Surgical antibiotic prophylaxis is rarely required for dental procedures.

In patients without profound immune compromise, surgical antibiotic prophylaxis is only indicated for some dentoalveolar procedures. Examples of procedures that do not require surgical antibiotic prophylaxis include third molar surgery, tooth extractions and insertion/removal of dental implants and peri-implant surgery. Examples of procedures that do require surgical antibiotic prophylaxis include intraoral bone grafting procedures, orthognathic surgery and cleft lip and palate repairs.

In patients with profound immune compromise, the need and regimen of surgical antibiotic prophylaxis is determined by the specialist managing the patient’s immune compromise.

The recommendation for prophylaxis where the incision is through the oral mucosa only is benzylpenicillin 1.2g intravenously within 1 hour before the incision. For procedures involving incision through the skin and oral mucosa, the recommendation is cefazolin 2g within 1 hour before the incision. Do not give additional doses once the procedure is completed. For the full recommendations see Therapeutic Guidelines Oral and Dental Version 4.

## Appendix Table 2: Definitions NAPS

## Appendix Table 3: Hospital and Surgical NAPS compliance with guidelines assessment criteria

| **Compliance with guidelines** (only choose one of the following six criteria) | |
| --- | --- |
| Compliant with Therapeutic Guidelinesa | The prescription complies with the current Therapeutic Guidelinesa, including:   - route, dose, frequency AND - takes into account acceptable alterations due to age, weight, renal function, allergies, other prescribed medications etc. |
| Compliant with locally endorsed guidelinesb | The prescription complies with an officially endorsed local guideline, including:   - route, dose, frequency AND - takes into account acceptable alterations due to age, weight, renal function, allergies, other prescribed medications etc.   This does not include individual, departmental, or historical guidelines that do not have executive or drug and therapeutic committee approval  If the local guidelines are based exactly on the Therapeutic Guidelinesa, then choose the ‘*Therapeutic Guidelines*’ in preference to ‘*Local guidelines*’ |
| Non-compliant with guidelines | There is non-compliance with both Therapeutic Guidelinesa and local guidelines. UNLESS  the prescription takes into account acceptable alterations due to age, weight, renal function, allergies, other prescribed medications etc. |
| Directed therapy | The prescription has changed from empiric to directed therapy with microbiology culture or susceptibility results available |
| No guidelines available | There are no guidelines available for the documented or presumed indication |
| Not assessable | The medical records are not comprehensive enough to determine a documented or presumed indication  OR  It is difficult to assess if there is compliance |

a. Antibiotic Expert Group. Therapeutic Guidelines: Antibiotic. Version 16 (2019). Melbourne <http://online.tg.org.au/ip/>^9^
b. Local guidelines must be authorised and readily available on wards or on the hospital intranet. They cannot be a web link to international guidelines or other non-approved sites. Exceptions include paediatric and neonatal guidelines from an Australian children’s hospital and links to other official guidelines within a hospital’s network.

## Appendix Table 4: Surgical NAPS appropriateness definition
